# Relationship between resilience, social support, and mental health in breast cancer patients based on the four-quadrant health model

**DOI:** 10.3389/fpubh.2026.1796977

**Published:** 2026-06-18

**Authors:** Shuangrui Sui, Weicong Ma, Zhihui Tan, Kai Feng, Weitao Yan, Xueying Su

**Affiliations:** 1 Breast Disease Diagnosis and Treatment Center, Qinhuangdao, Hebei, China; 2 Department of Allergy, Tangshan, Hebei, China

**Keywords:** breast cancer, four-quadrant health model, mental health, resilience, social support

## Abstract

**Background:**

Breast cancer is a major public health problem that exposes patients to persistent physical symptoms and psychological distress across diagnosis, treatment, and follow-up. Objective: This study explored the associations of resilience and social support with mental health indicators among individuals with breast cancer and examined whether a Four-Quadrant Health Model could distinguish clinically meaningful physical-psychological risk profiles.

**Methods:**

This secondary cross-sectional analysis used baseline data from a parent breast cancer care-management project. A convenience sample of 1,052 breast cancer inpatients and follow-up patients was analyzed. Measures included the Connor-Davidson Resilience Scale, Social Support Rating Scale, Hospital Anxiety and Depression Scale, Fear of Progression Questionnaire, and MD Anderson Symptom Inventory-Breast Cancer module. Primary analyses retained continuous scores for correlation and regression models. The four-quadrant classification was used as an additional clinical stratification framework based on physical symptom burden and HADS-defined psychological status. Pearson correlations, hierarchical multiple linear regression, and one-way ANOVA with robustness checks were used for statistical analysis.

**Results:**

Resilience showed a weak positive association with social support (*r* = 0.04) and significant negative associations with anxiety, depression, and fear of illness progression (*r* = −0.18 to −0.24, *p* < 0.001). Multiple linear regression showed standardized *β* coefficients of −0.26 for resilience and −0.08 for social support after adjustment for age, explaining 22.3% of the variance in mental distress (*F*(3, 1,048) = 100.3, *p* < 0.001). Significant differences in the mental distress composite were observed across the four quadrants (*F*(3, 1,048) = 565.8, *p* < 0.001, *η*^2^ = 0.62).

**Conclusion:**

Higher resilience and stronger social support were independently associated with lower psychological distress among breast cancer patients, with resilience showing the stronger association. The Four-Quadrant Health Model distinguished clinically meaningful risk profiles and may support psychosocial triage when used alongside continuous assessment. Because this was a secondary cross-sectional analysis based on convenience sampling from one tertiary hospital, the findings should be interpreted as associations and confirmed in longitudinal, multi-center studies.

## 1 Introduction

Breast cancer remains the most common malignancy among women worldwide, with increasing incidence rates in many countries (1). Improvements in diagnosis and treatment have increased survival for many patients (2), yet the physical and psychological burden associated with diagnosis, surgery, chemotherapy, radiotherapy, endocrine therapy, and survivorship frequently leads to persistent distress (3). Evidence suggests that anxiety and depression are common in women with breast cancer (4), and fear of recurrence or progression may persist for years after treatment completion (5).

The impact of breast cancer extends beyond physical symptoms and treatment side effects. Patients frequently face disruptions to self-image, family and social roles, intimate relationships, fertility expectations, fears about mortality, and concerns about treatment efficacy (3, 4). These challenges may impair quality of life and can influence treatment adherence and broader health outcomes (6, 7).

Frameworks that integrate physical and psychological dimensions of health are useful for understanding patient experiences and developing targeted psychosocial care. The Four-Quadrant Health Model used in the present study was operationalized from the biopsychosocial model and health-related quality-of-life frameworks, both of which emphasize that biological status, symptoms, emotional functioning, and social context jointly shape patient outcomes (8, 9). In this study, physical state and psychological state were treated as two clinically interpretable axes, allowing patients to be classified as Quadrant I (poor physical/poor psychological), Quadrant II (good physical/poor psychological), Quadrant III (good physical/good psychological), or Quadrant IV (poor physical/good psychological).

The rationale for using a four-quadrant framework is clinical rather than purely statistical. Converting continuous variables into categorical groups can reduce statistical power, increase residual confounding, and obscure within-group heterogeneity (10, 11). Therefore, the primary analyses in this study retained continuous resilience, social support, symptom, and distress scores. The quadrant classification was used as a supplementary triage-oriented framework to determine whether validated physical and psychological thresholds could identify subgroups with meaningfully different distress profiles. This approach preserves continuous-variable analyses while also providing a simple clinical language for risk stratification.

Within the broader literature on psychological adjustment to cancer, resilience and social support have emerged as two important protective factors. Psychological resilience, commonly defined as the capacity to adapt to stress or adversity, has been associated with better adjustment and quality of life among patients with cancer (12, 13). Resilience is thought to operate through mechanisms such as coping flexibility, meaning-making, positive emotion regulation, and maintenance of valued activities despite illness-related stress (14). However, the degree to which resilience is associated with specific psychological challenges in breast cancer, such as fear of progression, remains incompletely understood.

Social support has also been identified as a crucial resource for patients with cancer. The perception of reliable emotional, informational, and practical assistance is central to the stress-buffering model (15) and has been linked to adjustment during cancer diagnosis and treatment (16). Nevertheless, the strength and direction of associations between social support and specific mental health indicators vary across studies, and the relative importance of social support compared with resilience has received limited attention in breast cancer cohorts.

Few investigations have simultaneously examined how resilience and social support are associated with mental health indicators in breast cancer patients within an integrated physical-psychological stratification framework. Questions remain about their relative contributions, their potential independent associations with distress, and whether these associations differ across dimensions of psychological distress (17). Age-related differences in symptom burden and psychological distress have also been reported in oncology populations, suggesting that age should be considered when modeling patient distress (18).

Accordingly, there is a gap in studies that combine resilience, social support, and quadrant-based physical-psychological stratification in breast cancer populations. The present study addressed this gap in a large Chinese breast cancer cohort by using continuous statistical models for resilience and social support while also evaluating whether a four-quadrant clinical framework could discriminate psychological risk profiles. This dual analytic strategy was intended to avoid overreliance on categorization while retaining a clinically accessible method for screening and triage.

The current study aimed to (1) describe the relationships among resilience, social support, anxiety, depression, and illness progression fear; (2) evaluate the independent associations of resilience and social support with a composite measure of psychological distress after accounting for age; and (3) compare psychological distress and protective factors across the four physical-psychological quadrants.

## 2 Methods

### 2.1 Study design and participants

A secondary cross-sectional analysis was conducted using baseline questionnaire and medical-record data from the parent project titled Research on the Application of PERMA Positive Psychological Intervention in the Whole Process Management of Breast Cancer Patients. From January 2023 to June 2025, the parent study used convenience sampling to recruit 1,052 female breast cancer patients from the breast surgery department and outpatient follow-up center of a tertiary (Grade A) hospital in China. The mean age of participants was 50.8 ± 10.2 years. Inclusion criteria were: (1) confirmed diagnosis of breast cancer; (2) awareness of their cancer diagnosis; (3) age ≥ 18 years; and (4) ability to read and understand the questionnaires. Exclusion criteria included: (1) severe physical complications that would impede completion of assessments; (2) cognitive impairment or psychiatric disorders that would affect comprehension and response validity; and (3) concurrent diagnosis of other malignancies. Stage IV disease was not an automatic exclusion criterion; patients at any stage were eligible if they were clinically stable enough to complete the questionnaires and met the other criteria. All participants provided written informed consent for the parent-study questionnaire assessment and use of de-identified data for research. The study protocol was approved by the ethics committee of Qinhuangdao First Hospital (Approval Number: 2023wJ010) and was conducted in accordance with the Declaration of Helsinki. For the present anonymized secondary analysis of existing baseline data, the ethics committee waived the requirement for additional consent.

The recruitment procedure belonged to the parent study. During hospitalization or routine follow-up appointments, trained research assistants approached potentially eligible patients, explained the study purpose and procedures, and invited them to complete standardized questionnaires in a private setting. Assistance was available if needed, but research assistants were instructed only to clarify item wording and not to influence responses. Demographic and clinical information, including age, educational level, marital status, employment status, cancer stage, treatment modalities, and time since diagnosis, was obtained through structured interview and medical record review during the parent study. For the de-identified baseline analysis file used in this secondary analysis, age was retained as an analytic covariate; complete stage strata and detailed treatment categories were not retained in a format that supported reliable descriptive tabulation or subgroup modeling. The present analysis used the de-identified baseline dataset before any PERMA-based intervention activities were delivered.

Because the analysis was based on one tertiary hospital and a convenience sample from the parent project, sampling was not probability-based and hospital-level clustering could not be estimated. These design characteristics were addressed by standardized recruitment and data-collection procedures, by medical-record verification of clinical information, and by cautious interpretation of all findings as associations rather than causal effects.

### 2.2 Measurement instruments

#### 2.2.1 Resilience

Psychological resilience was assessed using the Chinese version of the Connor-Davidson Resilience Scale (CD-RISC) (12, 19). This 25-item instrument measures the ability to cope with stress and adversity across five dimensions: personal competence, trust in one’s instincts/tolerance of negative affect, positive acceptance of change/secure relationships, control, and spiritual influences. Participants rated each item on a 5-point Likert scale ranging from 0 (“not true at all”) to 4 (“true nearly all the time”), yielding a total score between 0 and 100, with higher scores indicating greater resilience. The Chinese version has demonstrated acceptable psychometric properties in Chinese populations (19). No diagnostic cutoff was applied to the CD-RISC in this study; scores were retained as continuous variables. In the current sample, Cronbach’s alpha was 0.93, indicating excellent reliability.

#### 2.2.2 Social support

The Social Support Rating Scale (SSRS) was used to evaluate perceived social support (20). This instrument, widely used in Chinese populations, comprises 12 items across three dimensions: objective support, subjective support, and utilization of support. The scale yields scores ranging from 12 to 66, with higher scores indicating stronger social support. Because the SSRS is intended to quantify the level of social support rather than to diagnose a clinical condition, no categorical cutoff was used; total scores were analyzed continuously. For the present sample, Cronbach’s alpha was 0.90.

#### 2.2.3 Anxiety and depression

The Hospital Anxiety and Depression Scale (HADS) was employed to assess symptoms of anxiety and depression (21). This 14-item scale consists of two 7-item subscales measuring anxiety (HADS-A) and depression (HADS-D), respectively. Each item is scored on a 4-point scale (0–3), resulting in possible scores of 0–21 for each subscale. Higher scores indicate greater symptom severity. Scores of 8–10 on either subscale were interpreted as borderline symptoms and scores ≥11 as probable clinical symptoms, consistent with commonly used HADS thresholds and validation evidence in cancer populations (22, 23). The Chinese version of HADS has shown acceptable psychometric performance (22). In this study, Cronbach’s alpha values were 0.87 for the anxiety subscale and 0.84 for the depression subscale.

#### 2.2.4 Fear of illness progression

Fear regarding cancer progression was measured using the short form of the Fear of Progression Questionnaire (FoP-Q-SF) (24). This 12-item instrument assesses concerns about disease advancement and its potential consequences. Items are rated on a 5-point Likert scale from 1 (“never”) to 5 (“very often”), yielding total scores between 12 and 60. Higher scores reflect greater fear of progression. The Chinese version of the FoP-Q-SF has demonstrated reliability and validity in women with breast cancer (25). Because the FoP-Q-SF was used as a continuous severity measure rather than a diagnostic instrument, no dichotomous cutoff was applied. In the current sample, Cronbach’s alpha was 0.89.

#### 2.2.5 Four-quadrant categorization

Participants were classified into one of four quadrants based on the Four-Quadrant Health Model. Physical state was assessed using the physical function and symptom burden component of the MD Anderson Symptom Inventory-Breast Cancer module (MDASI-BR), which measures the severity of common cancer-related symptoms and interference with daily activities (26, 27). Each symptom is scored on a 0–10 numeric scale (0 = no symptom, 10 = worst imaginable). Total scores were transformed to a 0–100 scale, with lower scores indicating better physical function. A score of ≥45 was selected *a priori* to indicate at least moderate symptom burden approaching the scale midpoint; this threshold identifies patients with clinically meaningful functional interference while remaining independent of the psychological outcome.

To avoid circularity in the analytic design, the psychological state axis was determined independently of the composite mental distress outcome variable. Psychological state was classified using the raw HADS total score (HADS-A plus HADS-D). A total score ≥16 was classified as poor psychological state because it reflects at least borderline symptoms across both subscales or probable symptoms on one subscale and is consistent with HADS screening evidence in cancer populations (21–23). The composite mental distress score, which integrates standardized *z*-scores of anxiety, depression, and fear of illness progression, incorporates a broader psychological construct and uses a continuous *z*-score transformation. Thus, the four possible categories were Quadrant I (poor physical/poor psychological), Quadrant II (good physical/poor psychological), Quadrant III (good physical/good psychological), and Quadrant IV (poor physical/good psychological).

#### 2.2.6 Mental distress composite

To create a comprehensive measure of psychological distress, standardized *z*-scores were calculated for anxiety, depression, and fear of progression scores. The arithmetic mean of these three *z*-scores was then computed to form a composite mental distress variable. This approach allowed anxiety, depressive symptoms, and fear of illness progression to contribute equally while retaining a continuous outcome. The composite score had a mean of 0 and a standard deviation of 0.84, with higher values indicating greater overall psychological distress. This composite variable is operationally distinct from the HADS-based psychological classification used for quadrant assignment because it includes fear of progression and applies *z*-score standardization, whereas the quadrant axis uses the raw HADS total score with a clinical threshold.

### 2.3 Statistical analysis

All analyses were undertaken in Python 3.11 (Pandas, SciPy, matplotlib). Descriptive statistics (mean, SD, range, skewness, and kurtosis) were calculated for all continuous variables, and missing data, outliers, and approximate normality were reviewed. Pearson correlation coefficients were calculated to examine relationships among resilience, social support, anxiety, depression, fear of illness progression, and the mental distress composite. Correlations greater than or equal to 0.10 were considered potentially meaningful in the context of psychosocial research.

Associations of resilience and social support with mental distress were analyzed with hierarchical multiple linear regression, controlling for age. Standardized coefficients (*β*), standard errors, *R*^2^, 95% confidence intervals, *t*-values, and *p-*values were reported. Model assumptions, including linearity, normality of residuals, and multicollinearity, were evaluated before interpretation. Given the cross-sectional nature of the data, regression coefficients reflect statistical associations rather than causal or directional effects; terms such as independent variable and outcome are used in the conventional statistical sense and do not imply temporal or causal precedence.

Differences among the four quadrant groups were examined with one-way ANOVA, with eta-squared (*η*^2^) reported as an effect size. *Post-hoc* pairwise comparisons were estimated using the least significant difference method for descriptive interpretability. Because quadrant group sizes were unequal, particularly for Quadrant IV (*n* = 30), Welch’s ANOVA was additionally conducted as a robustness check (28), and Games-Howell *post-hoc* tests were performed to account for unequal variances and unequal sample sizes (29). Because all participants were recruited from one hospital, no between-hospital random effect or hospital-level correlation parameter was estimable. Future multi-center studies should use mixed-effects models or generalized estimating equations to account for hospital-level clustering. An alpha level of *α* = 0.05 (two-tailed) was applied for all tests.

### 2.4 Strategies to minimize self-report bias

As all relevant psychological and functional variables were acquired through self-report questionnaires, several steps were taken to mitigate self-report bias, including mood-congruent memory bias and social desirability bias. First, verbal and written instructions were standardized for all participants, emphasizing independent understanding of item content and honest responses. Second, questionnaires were administered in a private, quiet space without family members or non-research medical personnel present. Third, all instruments had validated Chinese versions or established Chinese use. Fourth, available demographic and clinical information was cross-checked with electronic medical records when available to reduce reliance on recall. Fifth, trained research assistants clarified only item wording and did not suggest or influence answers.

## 3 Results

### 3.1 Descriptive statistics

Table 1 presents the descriptive statistics for the main study variables retained in the de-identified analysis file (*n* = 1,052). The mean resilience score was 76.42 (SD = 11.15), with a range from 38 to 98, indicating generally moderate to high levels of resilience in this sample. The distribution showed a slight negative skew (−0.62), suggesting a tendency toward higher resilience scores. Social support scores averaged 31.58 (SD = 12.43), ranging from 13 to 63, with a positive skew (0.82), indicating that fewer participants reported very high levels of social support.

**Table 1:** Descriptive statistics for main study variables (*n* = 1,052).

Variable	*n*	Mean	SD	Min	Max	Skew	Kurtosis
Resilience	1,052	76.42	11.15	38	98	−0.62	−0.28
Social support	1,052	31.58	12.43	13	63	0.82	−0.78
Anxiety	1,052	8.65	5.38	0	20	0.16	−1.44
Depression	1,052	8.17	5.82	0	23	0.72	−0.68
Illness fear	1,052	31.52	8.55	12	57	−0.06	0.18
Distress	1,052	0.00	0.84	−2.83	2.41	−0.03	−0.31

Distress = mental distress composite, calculated as the mean *z*-score of anxiety, depression, and illness fear.

For the negative emotional indices, the mean anxiety score was 8.65 (SD = 5.38), which is close to the clinical threshold of 8, suggesting that a substantial proportion of participants experienced clinically meaningful anxiety symptoms. Depression scores averaged 8.17 (SD = 5.82), also approaching clinical significance. Fear of illness progression showed a mean of 31.52 (SD = 8.55), with a relatively normal distribution (skewness = −0.06). The composite mental distress variable, by definition, had a mean of 0 (SD = 0.84) and ranged from −2.83 to 2.41, with an approximately normal distribution.

### 3.2 Bivariate correlations

Table 2 shows the Pearson correlation coefficients among the six key variables. Notably, resilience and social support demonstrated only a weak, non-significant positive correlation (*r* = 0.038, *p* = 0.216), suggesting these constructs represent largely independent protective resources in this population.

**Table 2:** Bivariate Pearson correlations among key variables (*n* = 1,052).

Var 1	Var 2	*r*	*p*
Resilience	Social support	0.038	0.216
Resilience	Anxiety	−0.241	<0.001
Resilience	Depression	−0.237	<0.001
Resilience	Illness fear	−0.183	<0.001
Resilience	Mental distress	−0.283	<0.001
Social support	Anxiety	−0.291	<0.001
Social support	Depression	−0.258	<0.001
Social support	Illness fear	0.195	<0.001
Social support	Mental distress	−0.093	0.003
Anxiety	Depression	0.778	<0.001
Anxiety	Illness fear	0.225	<0.001
Anxiety	Mental distress	0.791	<0.001
Depression	Illness fear	0.203	<0.001
Depression	Mental distress	0.784	<0.001
Illness fear	Mental distress	0.729	<0.001

Resilience showed significant negative correlations with all negative emotional indicators: anxiety (*r* = −0.241, *p* < 0.001), depression (*r* = −0.237, *p* < 0.001), and fear of illness progression (*r* = −0.183, *p* < 0.001). The correlation between resilience and the composite mental distress was stronger (*r* = −0.283, *p* < 0.001), indicating that higher resilience was consistently associated with lower psychological distress across multiple domains.

Social support demonstrated significant negative correlations with anxiety (*r* = −0.291, *p* < 0.001) and depression (*r* = −0.258, *p* < 0.001). Interestingly, social support showed a positive correlation with fear of illness progression (*r* = 0.195, *p* < 0.001), suggesting that patients with stronger social networks might experience greater concerns about disease progression. The overall correlation between social support and mental distress was negative but relatively weak (*r* = −0.093, *p* = 0.003).

Among the negative emotional indicators, anxiety and depression were strongly correlated (*r* = 0.778, *p* < 0.001), while both showed moderate correlations with fear of illness progression (*r* = 0.225 and *r* = 0.203, respectively, both *p* < 0.001). All three indicators were strongly associated with the composite mental distress measure (*r* = 0.729 to 0.791, all *p* < 0.001).

The complete bivariate correlation matrix is provided in Table 2, which was retained as the sole correlation display to avoid redundant presentation of the same information.

### 3.3 Multiple linear regression analysis of mental distress

Table 3 shows the results of the multiple linear regression analysis examining associations with mental distress. The overall model was statistically significant, *F*(3, 1,048) = 100.3, *p* < 0.001, and accounted for 22.3% of the variance in mental distress (*R*^2^ = 0.223).

**Table 3:** Multiple linear regression analysis of associations with mental distress (*R*^2^ = 0.223, *n* = 1,052).

Variable	*β*	SE	95% CI lower	95% CI upper	*t*	*p*
Intercept	0.000	0.036	−0.071	0.071	0.00	1.000
Resilience	−0.258	0.028	−0.313	−0.203	−9.11	<0.001
Social support	−0.085	0.031	−0.146	−0.024	−2.68	0.008
Age	−0.198	0.028	−0.253	−0.143	−7.05	<0.001

After controlling for age, both resilience (*β* = −0.258, 95% CI = −0.313 to −0.203, *p* < 0.001) and social support (*β* = −0.085, 95% CI = −0.146 to −0.024, *p* = 0.008) were independently and negatively associated with mental distress. In practical terms, a one standard deviation increase in resilience was associated with approximately a 0.26 standard deviation decrease in mental distress, and social support showed a comparatively smaller but statistically significant association of 0.09 standard deviation. Age was also significantly and negatively associated with mental distress (*β* = −0.198, 95% CI = −0.253 to −0.143, *p* < 0.001), indicating that older participants reported lower levels of distress. The relative magnitude of these associations suggests resilience enhancement as a primary psychosocial target, while social support strengthening may serve as an additional strategy.

**Figure 1:**
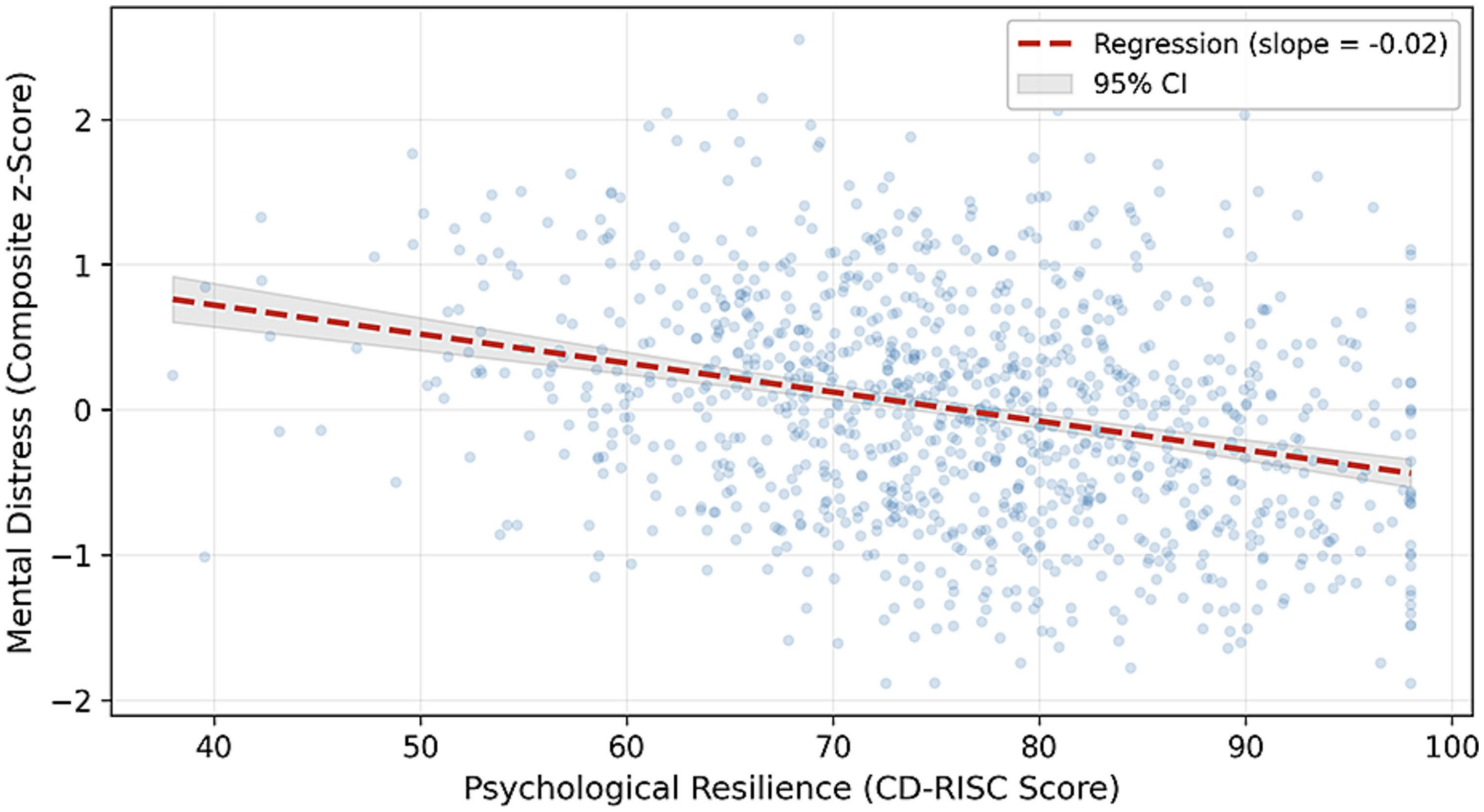
Scatter plot illustrating the negative association between psychological resilience and the mental distress composite in breast cancer patients (unstandardized slope = −0.02; standardized *β* = −0.258, *p* < 0.001; *n* = 1,052).

Figure 1 demonstrates the negative association between resilience and the mental distress composite. The dashed regression line represents the unstandardized linear slope (slope = −0.02), while the standardized association is reported in Table 3 (*β* = −0.258). The gray shading represents the 95% confidence band. Each point reflects an individual patient (*n* = 1,052).

### 3.4 Quadrant-based comparisons

The distribution of participants across the quadrants was as follows: 254 individuals (24.1%) in Quadrant I (poor physical status/poor psychological status), 441 individuals (41.9%) in Quadrant II (good physical status/poor psychological status), 327 individuals (31.1%) in Quadrant III (good physical status/good psychological status), and 30 individuals (2.9%) in Quadrant IV (poor physical status/good psychological status) (Table 4). Although the expanded sample of 1,052 participants improved statistical power across all subgroups, the Quadrant IV group (*n* = 30) remained relatively small, warranting some interpretive caution for pairwise comparisons involving this group. The small size of Quadrant IV likely reflects the clinical reality that maintaining positive psychological status in the presence of significant physical symptom burden is inherently difficult and relatively uncommon in this population.

**Table 4:** Mental distress composite *z*-scores across quadrant categories.

Quadrant	*n*	Mean ± SD
I (Phys−/Psych−)	254	0.91 ± 0.58
II (Phys+/Psych−)	441	0.29 ± 0.52
III (Phys+/Psych+)	327	−0.73 ± 0.40
IV (Phys−/Psych+)	30	−0.60 ± 0.40

**Figure 2:**
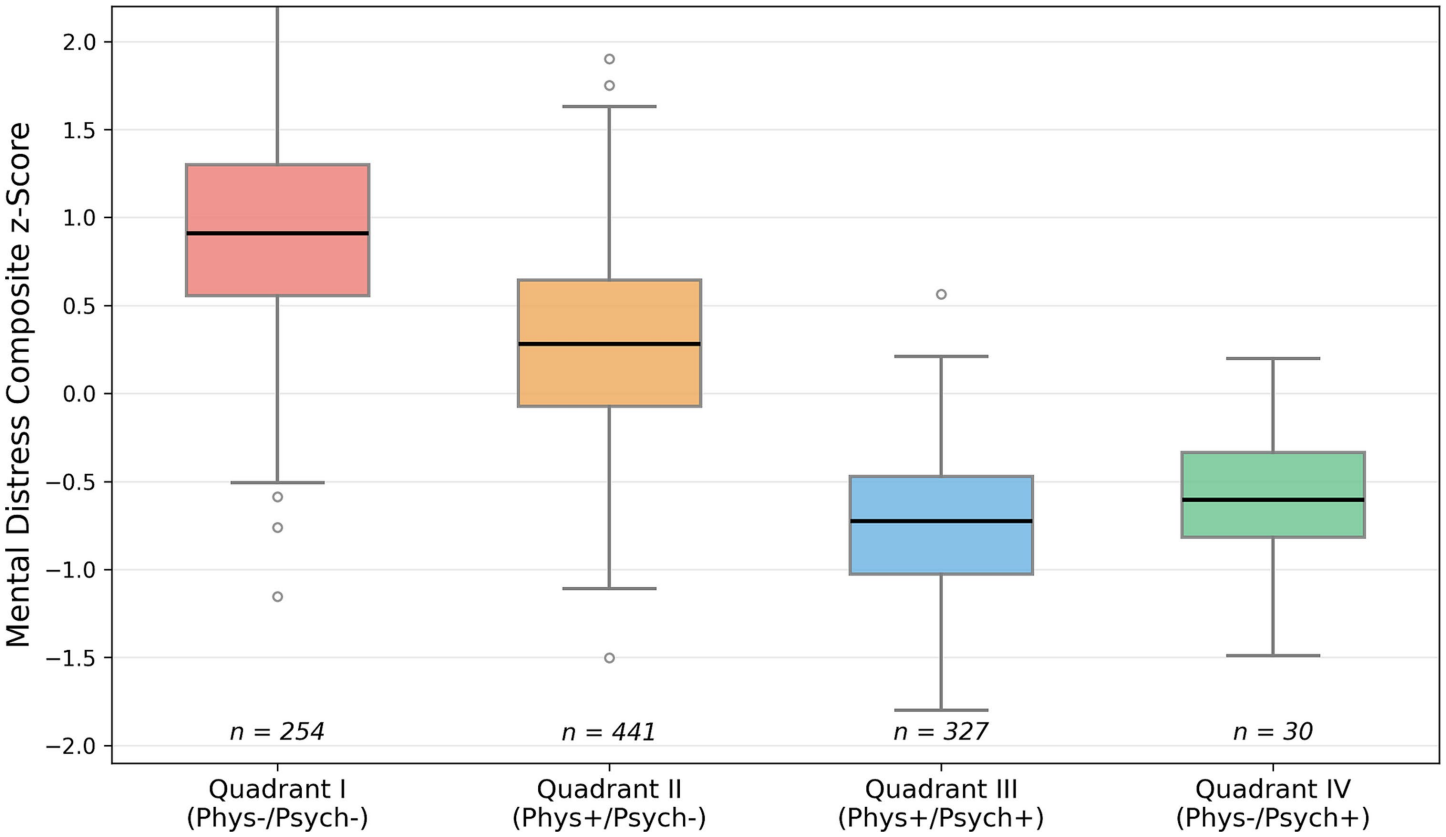
Box plot comparison of the mental distress composite across the four quadrants of the health model, ordered from left to right as Quadrant I, Quadrant II, Quadrant III, and Quadrant IV.

Figure 2 displays the distribution of the mental distress composite across the four quadrant categories in the order Quadrant I, Quadrant II, Quadrant III, and Quadrant IV. The central line marks the median, boxes represent the interquartile range, and whiskers denote 1.5 × IQR. Outliers are plotted as open circles.

The standard one-way ANOVA showed a statistically significant and large between-group effect of quadrant category on the mental distress composite (*F*(3, 1,048) = 565.78, *p* < 0.001, *η*^2^ = 0.62). As a robustness check addressing unequal group sizes, Welch’s ANOVA also yielded consistent results (Welch’s *F*(3, 137.3) = 619.41, *p* < 0.001). The effect size indicates that quadrant classification accounted for a substantial proportion of variance in mental distress. *Post hoc* analyses, supplemented by Games-Howell tests, showed that Quadrant I had the highest distress, Quadrant II had intermediate distress, and Quadrants III and IV had comparatively low distress. The difference between Quadrants III and IV was small and should be interpreted cautiously because of the smaller Quadrant IV sample (*n* = 30).

For clinical interpretation, Table 5 summarizes resilience, social support, anxiety, depression, fear of illness progression, and the mental distress composite across the four quadrants. The pattern indicates that combined poor physical and psychological status identifies the subgroup with the greatest distress burden, whereas good psychological status is associated with lower distress even when physical symptom burden is present.

**Table 5:** Means and standard deviations of study variables across quadrants.

Variable	I (*n* = 254)	II (*n* = 441)	III (*n* = 327)	IV (*n* = 30)
Resilience	74.18 ± 10.92	76.85 ± 10.88	79.15 ± 9.32	78.25 ± 8.65
Social support	26.12 ± 10.38	30.95 ± 11.52	35.42 ± 12.18	34.08 ± 11.25
Anxiety	14.85 ± 2.82	10.62 ± 2.98	4.52 ± 2.38	5.15 ± 2.44
Depression	15.28 ± 4.79	11.15 ± 4.41	3.58 ± 2.35	4.38 ± 2.58
Illness fear	34.47 ± 7.85	31.38 ± 6.21	26.22 ± 6.15	27.25 ± 5.82
Mental distress	0.91 ± 0.58	0.29 ± 0.52	−0.73 ± 0.40	−0.60 ± 0.40

## 4 Discussion

This study examined the associations among psychological resilience, social support, and mental health indicators in a sample of 1,052 breast cancer patients using the Four-Quadrant Health Model as an organizing framework. The results provide insight into the interplay of protective factors and psychological distress in this population and offer preliminary empirical support for the clinical utility of a quadrant-based physical-psychological screening approach.

Our results support the protective role of resilience and social support in breast cancer patients’ mental health, with several important nuances. Resilience appeared to be the more robust protective factor, showing consistent negative associations with anxiety, depression, fear of illness progression, and the mental distress composite. This aligns with previous findings suggesting that resilience is associated with maintained well-being when facing a cancer diagnosis and treatment (13, 14). These associations may reflect coping flexibility, positive emotion regulation, active coping, meaning-making, and reconstruction of cancer-related experiences.

Although resilience and social support had a weak and non-significant correlation (*r* = 0.04), indicating that they were primarily independent protective resources, theoretical perspectives often suggest that supportive relationships can facilitate resilience development (15, 16). In the present sample, internal psychological resources and coping capacities appeared to have a stronger association with distress than external social resources. The relationship between social support and psychological outcomes was more complex than expected. Although social support showed expected negative associations with anxiety and depression, it was positively correlated with fear of illness progression. This finding may reflect concern about the impact of disease progression on family members, increased illness-related communication in active support networks, or greater support-seeking among patients who already experience stronger fear.

Age was significantly associated with lower psychological distress, as older patients reported lower distress than younger patients after adjustment for resilience and social support. These results are consistent with evidence suggesting that older oncology patients may report lower distress for some psychological symptoms than younger patients, possibly because of accumulated life experience, altered health expectations, and improved emotion regulation across the lifespan (18).

The analysis of the Four-Quadrant Model demonstrated meaningful discriminant validity in this population. The model identified four subgroups with substantially different levels of the mental distress composite. Patients in Quadrant I (poor physical/poor psychological) had the highest distress, and patients in Quadrant III (good physical/good psychological) had the lowest distress. This pattern supports the view that integrated assessment of physical symptom burden and psychological status can improve risk stratification beyond either dimension alone (8, 9). The results also indicate that psychological state may be more strongly associated with overall distress risk than physical state alone. Patients with good psychological status had relatively low distress even when physical status was poor (Quadrant IV), while those with poor psychological status had greater distress even when physical status was good (Quadrant II).

Meaningful patterns in protective factors were also observed across the quadrants. Resilience and social support were lowest in Quadrant I and highest in Quadrant III, suggesting that these resources may be associated not only with lower distress but also with more favorable psychological status. The relatively small number of patients in Quadrant IV (*n* = 30) may reflect the difficulty of maintaining good psychological status when physical symptom burden is high. Although the total sample size of 1,052 improved overall statistical precision, the 2.9% representation of Quadrant IV indicates that findings involving this subgroup should be interpreted cautiously pending replication.

These findings underscore the value of assessing resilience and social support as part of routine psychosocial screening in breast cancer. Because resilience and social support were largely independent, assessment of both domains may provide more complete information than either domain alone. In post-surgical and follow-up care, resilience-building approaches and structured psychosocial interventions may be useful adjuncts to usual care, although intervention effects require prospective evaluation (30, 31). The complex association between social support and fear of progression suggests that clinical assessment should consider not only the quantity of available support but also the quality, emotional tone, and illness-related content of supportive interactions.

The Four-Quadrant Model may also function as a practical screening and triage tool in regular clinical care. The large between-group differences in distress indicate that the classification could help identify patients who may need higher-intensity psychosocial services. Patients in Quadrant I may require combined symptom management and psychological support, whereas those in Quadrant II may particularly benefit from psychological intervention despite relatively better physical status. Age differences across quadrants also suggest that younger patients may warrant heightened psychosocial attention, especially for concerns related to disease progression, fertility, family responsibilities, and role disruption (32).

Several limitations of this study should be acknowledged. First, the cross-sectional design prevents causal inferences from being drawn between protective factors and psychological outcomes; all reported associations are correlational, and the direction of effects cannot be established without longitudinal data. Second, the single-center convenience sample limits internal and external validity. Patients recruited from a tertiary hospital in Northern China may differ from those receiving care in community-level hospitals or rural settings, and the design did not permit modeling of hospital-level correlation. Future studies should use multi-center, stratified, cluster-based, or probability-based quantitative sampling and should apply mixed-effects models or generalized estimating equations when participants are nested within hospitals. Third, although cancer stage and treatment modality were reviewed during parent-study recruitment and stage IV disease was not an automatic exclusion criterion, stage distribution and treatment-stratified descriptive results could not be reliably reported because complete stage strata and detailed treatment categories were not retained in the de-identified analysis file used for this secondary analysis; future datasets should preserve full staging and treatment information to permit stage-adjusted and treatment-adjusted analyses. Fourth, self-report measures are vulnerable to mood-consistent memory bias, social desirability bias, and recall bias, although standardized instructions, private administration, validated instruments, and medical-record verification of available clinical information were used to reduce these risks. Fifth, although the psychological classification axis and the mental distress outcome were operationally distinct, partial conceptual overlap remains because both include HADS anxiety and depression components; future studies should use entirely independent instruments for classification and outcome assessment. Sixth, the Quadrant IV group was small (*n* = 30), which limits precision for this subgroup. Finally, although categorization may support clinical communication, it can reduce information compared with continuous-variable modeling (10, 11); therefore, quadrant assignment should be used as an adjunct to, not a replacement for, continuous psychosocial assessment.

In this study, psychological resilience (*β* = −0.26) and social support (*β* = −0.09) were independently associated with lower mental distress in breast cancer patients, with resilience showing the stronger association. Age (*β* = −0.20) was also significantly associated with lower distress. The Four-Quadrant Health Model distinguished meaningful risk profiles, particularly identifying patients in Quadrant I (poor physical/poor psychological) as the subgroup with the highest distress. These findings support integrating resilience screening, social support assessment, and quadrant-based triage into breast cancer psychosocial care. Longitudinal, multi-center research is needed to confirm these associations, incorporate complete clinical staging data, and evaluate the effectiveness of quadrant-specific intervention strategies.

## Data Availability

The original contributions presented in the study are included in the article/supplementary material, further inquiries can be directed to the corresponding authors.
